# How the Cost of Purulent Ophthalmia in New York State Might Be Lessened1Address of the President at the seventy-seventh annual meeting of the Medical Society of the County of Erie, 1898.

**Published:** 1898-03

**Authors:** Lucien Howe

**Affiliations:** Buffalo, N. Y.


### HOW THE COST OF PURULENT OPHTHALMIA IN NEW YORK STATE MIGHT BE LESSENED.[1]

By LUCIEN HOWE, M. D., Buffalo, N. Y.

ABSTRACT of an address on the reasons for a law in New York state requiring the use of silver nitrate solutions for the prevention of purulent ophthalmia of infancy in public institutions.

| | |
|---|---|
| Total number of blind in New York state in 1890 was....... | 4,389 |
| 10.8 per cent. of these (Magnus) are due to ophthalmia of infancy, or say 10 per cent.; there are from this disease in this state over .................................... | 438 |

THE COST.

| | |
|---|---|
| If 8 per cent. can work, the number to be supported is over.. | 403 |
| Of this number 64 are in two schools in New York city and Batavia and their cost is.............................. | $16,547.38 |
| Cost for remaining 339, if all are paupers, would be........ | 42,375.00 |
| Or for the 403 victims of this disease the minimum cost to the state is known to be at least..................... | $ 58,922.38 |
| Probable annual cost is over ........................... | 110,000.00 |

1. Address of the President at the seventy-seventh annual meeting of the Medical Society of the County of Erie, 1898.

(1) This suffering for the individuals and cost to the state could be very greatly lessened by the use of the Credé method—namely, a 2 per cent. solution of silver nitrate. This fact is absolutely proved by the combined experience of obstetricians.

The evidence is furnished (*a*) by *published* statistics. See tables by Kostling, of Halle, giving the results published by obstetricians in various countries up to 1896. This shows that in

| | | | |
|---|---|---|---|
| 17,767 | births with | no treatment, 9.2 per cent of ophthalmia of infancy. | |
| 24,724 | " " | 2 per cent. solution of silver nitrate | 0.65 per cent. |
| 1,223 | " " | 1 per cent. solution of silver nitrate | 2.4 " " |
| 1,623 | " " | carbolic acid solutions | 7.7 " " |
| 965 | " " | 0.1 per cent. solution of sublimate | 0.6 " " |
| 1,396 | " " | other sublimate solutions | 0.4 " " |
| 6,155 | " " | sterilised water | 2.8 " " |
| 701 | " " | iodide trichloride solutions | 1.2 " " |

Evidence for the Credé method furnished also (*b*) by the *unpublished* experience of leading obstetricians in this state, who have said that Credé's method should be made obligatory in public institutions. Among those in the city of New York, for example, are Drs. Boldt, Brettauer, Byrne, T. W. Cleaveland, Clement Cleaveland, Coe, Currier, Gibb, Emmett, Goffe, Jarman, Jacobi, Marx, McLean, Morrill, Munde, A. M. Thomas, T. C. Thomas, Tucker, Tuttle, Vineberg, Von Ramdohr, Wiener and other obstetricians equally prominent, in other portions of the state.

(2) Silver nitrate solution is practically free from danger. Among the fifty odd thousand cases, referred to above, and among the many more thousand in which the method has been used, only four cases have occurred which give even the suspicion of danger. In these the evidence is questionable, or the results simply inconvenient, but not serious.

(3) Two per cent. silver nitrate solution is not necessarily painful, if preceded by cocaine, properly applied.

(4) In spite of all this evidence in its favor silver nitrate solution is not used as it should be, especially in county almshouses.

This last is shown by replies to letters on this point (*a*) from fifty-eight hospitals in this state, and (*b*) from forty-six almshouses in this state. These letters show that this preventive treatment is used less in the rural districts than it is in or near the large centers of population in this state. The census of 1890 also shows that blindness is more common in those rural districts than in or near the large centers of population. These two facts are apparently related to each other.

The foregoing facts show that the number of blind from this disease is large, that its cost is great, that the combined experience of obstetricians, as published by observers in different parts of the world, point to one remedy as much better than any other thus far known, a remedy that is safe, easy of application and, if generally used, would greatly reduce the number of blind and the consequent cost to the state. Such statistics alone indicate the desirability of having that treatment made compulsory. In addition, however, there are other reasons, not statistical, in favor of such a law. They are:

(*a*) That when the state assumes the care of a child by supporting it, the child becomes practically its ward. In that case the state, as the guardian, has the right to dictate what treatment shall be employed for the child and if one method of treatment has been proved by vast experience to be superior to every form of treatment thus far known, then the state has the right to demand that any physician treating such a child shall use that form of treatment which insures the greatest amount of safety.

(*b*) In the same manner it is not only the right, but it is also the duty of the state to demand such treatment for pauper children, this being not simply for the sake of the child, but also to relieve the state from heavy and, to a great extent, unnecessary taxation.

(*c*) The state has already established this principle of compelling physicians to follow certain procedures, not only by obliging them to report contagious diseases and imposing other duties upon them, but especially has it done so by the enforcement of vaccination. In this it has furnished a precedent for compelling physicians to use a certain method of treatment to prevent a certain disease.

The result of such a law would be:

1. First, that it would immediately tend to lessen the number of children thus affected.

2. The indirect effect would be good in sustaining practitioners who do use this method in spite of objectors.

3. The indirect effect would also be good in condemning obstetricians who neglect its use and, by such omission, run greater risks of adding to the number of blind children, nearly every one of whom, whether paupers or not, must be educated at the expense of the state.

183 Delaware Avenue.

